# Prämenstruelles Syndrom und prämenstruelle dysphorische Störung – Übersicht zu Pathophysiologie, Diagnostik und Therapie

**DOI:** 10.1007/s00115-024-01625-5

**Published:** 2024-02-23

**Authors:** Jana Haußmann, M. Goeckenjan, R. Haußmann, P. Wimberger

**Affiliations:** 1 Klinik und Poliklinik für Gynäkologie und Geburtshilfe, Universitätsklinikum Carl Gustav Carus an der Technischen Universität Dresden, Fetscherstr. 74, 01307 Dresden, Deutschland; 2 Klinik und Poliklinik für Psychiatrie und Psychotherapie, Universitätsklinikum Carl Gustav Carus an der Technischen Universität Dresden, Fetscherstr. 74, 01307 Dresden, Deutschland

**Keywords:** Prämenstruelles Syndrom, Prämenstruelle dysphorische Störung, Lutealphase, Ovarielle Steroidhormone, Depression, Premenstrual syndrome, Premenstrual dysphoric disorder, Luteal phase, Ovarian steroids, Depression

## Abstract

Beim prämenstruellen Syndrom und der prämenstruellen dysphorischen Störung handelt es sich um episodisch in der 2. Zyklushälfte auftretende psychische und physische Symptome mit relevanter sozialer und beruflicher Beeinträchtigung im Alltag. Assoziierte depressive Symptome umfassen Stimmungsschwankungen, Depressivität und Gereiztheit, weshalb affektive Störungen eine relevante Differenzialdiagnose darstellen. Etwa 3–8 % aller Frauen im gebärfähigen Alter leiden an einem prämenstruellen Syndrom, während etwa 2 % von einer prämenstruellen dysphorischen Störung betroffen sind. Es werden genetische und soziobiografische Risikofaktoren diskutiert. Darüber hinaus werden genetische Polymorphismen spezifischer Hormonrezeptoren als ursächlich angenommen. Pathophysiologisch zentral scheint eine komplexe Interaktion von zyklischen hormonellen Veränderungen und dem zentralen Neurotransmitterhaushalt zu sein. Ein Ungleichgewicht der Wirkungen von Östrogen und Progesteron in der Lutealphase wird als endokrine Ursache der Symptomatik angenommen. Aus diesem Grunde wird häufig ein initialer Therapieversuch mittels Progesteronsubstitution in der zweiten Zyklushälfte durchgeführt, wobei die Evidenz diesbezüglich begrenzt ist. Auch die Gabe oraler kombinierter Kontrazeptiva stellt eine Option dar. Insbesondere für die Behandlung mit selektiven Serotoninwiederaufnahmehemmer (SSRI) existieren zahlreiche Wirkbelege. In schweren Fällen kann die Gabe von GnRH(Gonadotropin-Releasing-Hormon)-Analoga mit Add-back-Therapie erwogen werden. Insbesondere im Bereich affektiver Störungen stellen prämenstruelle Syndrome klinisch relevante Differenzialdiagnosen und Komorbiditäten dar, die Behandler vor besondere klinische Herausforderungen stellen. Diese Übersichtsarbeit soll der Leserschaft daher eine klinische Orientierung im Umgang mit diesem Störungsbild geben.

## Abstract (en)

Premenstrual syndrome and premenstrual dysphoric disorder become episodically manifest during the second half of the female menstrual cycle and are characterized by psychological and physical symptoms causing relevant functional and social impairments. Mood swings, depression and dysphoria are associated depressive symptoms. Therefore, affective disorders should be considered as a differential diagnosis. Of women in reproductive age 3–8% suffer from premenstrual syndrome and 2% of women are affected by premenstrual dysphoric disorder. Genetic and sociobiographical risk factors are discussed. Furthermore, genetic polymorphisms of specific hormone receptors are considered to be genetic risk factors. From a pathophysiological perspective premenstrual syndrome and premenstrual dysphoric disorder are caused by a complex interaction between cyclic changes of ovarian steroids and central neurotransmitters. An imbalance of estrogen and progesterone in the luteal phase is believed to cause the symptoms. Therefore, the first treatment approach consists of regulation of the menstrual cycle or luteal support with progesterone or synthetic progestins even if their effectiveness has not yet been proven in randomized controlled studies and meta-analyses. The administration of combined oral contraceptives is also an option. Especially treatment with selective serotonin reuptake inhibitors (SSRI) represent an evidence-based approach. In severe cases the administration of gonadotropin releasing hormone (GnRH) agonists with add back treatment can also be considered. In the field of affective disorders premenstrual syndromes represent clinically relevant differential diagnoses and comorbidities, which confront the treating physician with particular clinical challenges. Therefore, this literature review gives the readership a clinical orientation for dealing with these disorders.

## Hintergrund

Das prämenstruelle Syndrom (PMS) und die ausgeprägtere Form, die prämenstruelle dysphorische Störung (PMDS), sind durch episodisch in der zweiten Zyklushälfte und typischerweise bis zum Beginn der Menstruation anhaltendende physische und psychische Symptome und Verhaltensänderungen charakterisiert, die in Summe zu relevanten Beeinträchtigungen des sozialen und beruflichen Funktionsniveaus führen. Häufige körperliche Symptome sind ein geblähtes Abdomen, Spannungsgefühl in der Brust und Kopfschmerzen, wobei auch funktioneller typischerweise vorrangig die psychischen Symptome beeinträchtigungsrelevant sind [1]. Diese umfassen insbesondere affektive Symptome wie Stimmungsschwankungen, Depressivität, Irritabilität und eine dysphorische Gereiztheit [2]. Hinsichtlich hereditärer Risikofaktoren werden genetische Polymorphismen im ESR1-Gen diskutiert, das für den Östrogenrezeptor‑α kodiert [3, 4]. Bezüglich weiterer Risikofaktoren besteht eine große Überlappung zu soziobiografischen Parametern, die auch Risikofaktoren für die Entstehung von affektiven Störungen und Angsterkrankungen sind [5]. Zu nennen sind hier ein geringes Bildungsniveau, Traumatisierung und vorbestehende Angststörungen [5]. Aktuelle Daten zeigen außerdem, dass Frauen mit Neigung zu ausgeprägtem stresshaftem Erleben (OR = 4,90; 95 %-CI: 2,70–8,89) und hohen Neurotizismuswerten (OR = 8,05; 95 %-CI: 3,07–2,12) häufiger an PM(D)S leiden [6]. Nikotinabusus, Ernährung mit höherem Anteil an Fett, Protein, Salz und Kaffee sowie Unter- oder Übergewicht sind als weitere, modifizierbare Risikofaktoren für das PMS/die PMDS bekannt [7].

## Methodik

Diese Übersichtsarbeit wurde unter Nutzung der über PubMed verbundenen biomedizinischen Datenbanken erstellt. Eine zusätzliche Suche erfolgte anhand der in den Literaturverzeichnissen gefundenen Original- und Übersichtsarbeiten. Dabei wurden präferenziell Originalarbeiten gesucht. Auch die verfügbaren Leitlinien wurden berücksichtigt. Die Stichwörter bei der Onlineliteraturrecherche waren: „premenstrual syndrome AND therapy“, „premenstrual dysphoric disorder AND therapy“, „premenstrual syndrome AND study“, „premenstrual dysphoric disorder AND study“ und „premenstrual syndrome AND review“, „premenstrual dysphoric disorder AND review“. In Anbetracht der schmalen Datenlage fanden neben verschiedenen Studiendesigns auch Literaturübersichten Berücksichtigung.

## Epidemiologie

Eine diagnosewertige PMS-Symptomatik betrifft 3–8 %, eine klinische relevante PMDS-Symptomatik etwa 2 % aller Frauen [1, 8]. Die unzureichend konsequente Anwendung diagnostischer Kriterien führte in früheren Arbeiten annehmbar zu einer erheblichen Überschätzung der PM(D)S-Prävalenz [9].

## Diagnostik

**Figure 1:**
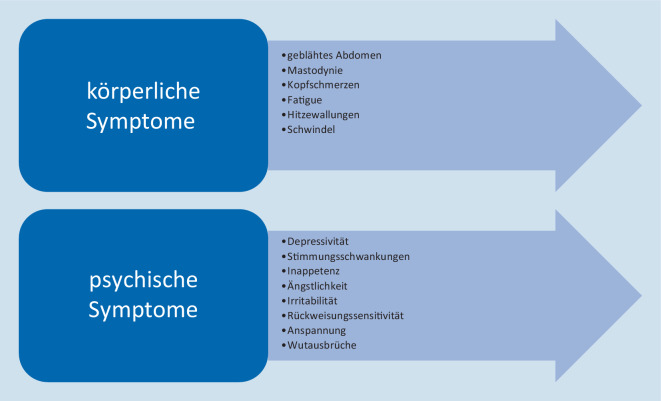

Die meisten Frauen im reproduktionsfähigen Alter haben ein bis zwei Tage vor der Menstruation milde emotionale und körperliche Symptome, die jedoch in aller Regel nicht beeinträchtigend und daher vom PMS abzugrenzen sind [10]. Grundsätzlich ist das PMS definiert als mindestens ein Symptom mit relevanter sozialer oder beruflicher Funktionsbeeinträchtigung mit Symptombeginn innerhalb von fünf Tagen vor der Menstruation [10]. Bei den Symptomkomplexen PMS und PMDS handelt es sich insgesamt um ein weites Spektrum emotionaler, behavioraler und kognitiver Symptome mit Beginn in der Lutealphase und prompter Remission in der Follikelphase [10]. Die häufig im Vordergrund stehenden affektiven Symptome umfassen Depressivität und Stimmungsschwankungen, Irritabilität, Ängstlichkeit, Gefühle von Anspannung, Wutausbrüchen, Appetitsteigung bis hin zu Essattacken, Rückweisungssensitivität und einen Interessenverlust [11]. Charakteristische körperliche Symptome sind eine Mastodynie, Kopfschmerzen, ein geblähtes Abdomen, eine Fatigue-artige Symptomatik und Hitzewallungen (Abb. 1; [11]). Die Symptomdauer beträgt durchschnittlich sechs Tage pro Monat mit Beschwerdemaximum vier Tage vor bis drei Tage nach Menstruationsbeginn [10, 11].

Das PMS ist mit einer signifikanten Lebensqualitätseinbuße assoziiert und es bestehen sogar Assoziationen zu suizidalem Verhalten [10, 12]. Möglicherweise ist auch das Risiko für das Auftreten depressiver Erkrankungen in der Menopause erhöht [13]. Die Unterbrechung des ovariellen Zyklus, beispielsweise in der Schwangerschaft oder postmenopausal, führt zu einem Sistieren des PMS [10].

## Diagnostische Kriterien

Gemäß allgemeiner diagnostischer Kriterien kann die Diagnose eines PMS gestellt werden, wenn 1 bis 4 Symptome vorliegen, von denen mindestens ein Symptom dem affektiven Spektrum zuordenbar ist oder wenn > 5 Symptome behavioraler oder somatischer Art vorliegen [10]. Wenn ≥ 5 Symptome vorliegen und eines davon dem affektiven Symptomspektrum zuordenbar ist, kann die Diagnose einer PMDS gestellt werden. Gemäß den Kriterien der ACOG (American College of Obstetricians and Gynecologists) kann ein PMS bereits diagnostiziert werden, wenn ein relevant funktionsbeeinträchtigendes Symptom mit Manifestation in der Lutealphase vorliegt [10]. Nach den ISPMD-Kriterien (International Society for Premenstrual Disorders) bedarf es für die Diagnosestellung mindestens eines affektiven oder behavioralen Symptoms, welches relevant funktionsbeeinträchtigend ist, mit Menstruationsbeginn oder kurz danach remittiert und von einem symptomfreien Intervall gefolgt ist [10].

In den ICD-10-Kriterien der WHO sind das PMS und die PMDS nicht im Abschnitt „Psychische und Verhaltensstörungen (F00-F99)“ aufgeführt, sondern werden als „N94,3 Prämenstruelle Beschwerden“ kodiert. Demnach wird das PMS als komplexe emotionale und körperliche Beschwerdesymptomatik beschrieben, die im Zusammenhang mit dem Menstruationszyklus steht, sich in jedem Monatszyklus 4 bis 14 Tage vor Menstruationsbeginn manifestiert und mit Beginn der Menstruation sistiert.

Die diagnostischen Kriterien einer PMDS hingegen werden im DSM‑5 (Diagnostic and Statistical Manual of Mental Disorders, Fifth Edition) definiert. Nach DSM-5-Kriterien kann die Diagnose eines PMDS gestellt werden, wenn behaviorale und körperliche Symptome für den Großteil des vorangegangenen Jahres bestanden und ≥ 5 Symptome in der Woche vor Menstruationsbeginn manifest sind, welche innerhalb weniger Tage nach Menstruationsbeginn vollständig remittieren. In den diagnostischen Kriterien wird auch darauf verwiesen, dass die PMDS möglicherweise eine zugrunde liegende psychiatrische Erkrankung überlagern kann, ohne jedoch eine bloße Exazerbation dieser darzustellen [10]. In Analogie dazu ist die PMDS-Diagnose auch im 2022 in Kraft getretenen ICD-11 enthalten.

**Figure 2:**
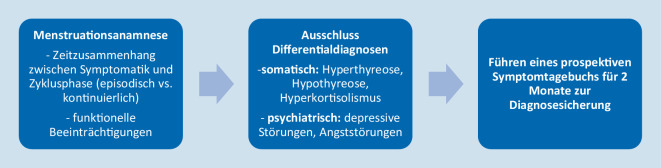

## Diagnostische Assessment

Das diagnostische Assessment bei PMS bzw. PMDS sollte vorrangig eine ausführliche Menstruationsanamnese mit Fokus auf dem Zeitzusammenhang zwischen Symptomatik und entsprechender Zyklusphase sowie die Erfassung von Symptombeginn, Symptomende, Symptomschwere und assoziierten funktionellen Beeinträchtigungen beinhalten [10]. Die Anamneseerhebung kann bei Patientinnen mit irregulärem Zyklus erschwert sein. Empfohlen wird der Ausschluss endokrinologischer Erkrankungen mit ähnlicher Beschwerdesymptomatik wie Hyper- und Hypothyreose sowie Hyperkortisolismus [10]. Beim Vorliegen einer mit einem PMS zu vereinbarenden klinischen Symptomatik wird das Führen eines prospektiven Symptomtagebuchs für zwei Monate zur Diagnosesicherung empfohlen. Symptomtagebücher können insbesondere helfen, den zyklischen Symptomcharakter in Bezug zum Zyklusverlauf herauszustellen, womit auch die diagnostische Abgrenzung zu kontinuierlich vorhandenen affektiven Symptomen in Form einer Dysthymie oder depressiven Erkrankungen unterstützt wird [10]. Zahlreiche Daten belegen, dass die diagnostische Zuverlässigkeit durch eine strukturierte Symptomerfassung verbessert wird [14]. Diesbezüglich ist jedoch kritisch zu diskutieren, dass dies für die Betroffenen eine hohe Belastung darstellt und die Behandlung nochmals um Monate hinauszögert, weshalb Symptomtagebücher in der Praxis kaum Anwendung finden und die Diagnostik vorwiegend anhand retrospektiver Aussagen erfolgt [14]. Ein Zyklusblatt als Monatsübersicht über mögliche Symptome kann beispielsweise über die Website der IAMPD (International Association for Premenstrual Disorders; https://iapmd.org/provider-resources) heruntergeladen werden. Darüber hinaus sollte das diagnostische Assessment durch strukturierte klinische Interviews (z. B. SCID-5-CV, SCID-PMMD) sowie geeignete Selbstfragebögen (z. B. PSST) ergänzt werden [15–17] (Abb. 2).

## Pathophysiologie

Die Entwicklung eines PMS ist gemäß aktueller Evidenz als Folge einer komplexen Interaktion zwischen zyklischen Veränderungen ovarieller Hormone in der Lutealphase und dem zentralen Neurotransmitterhaushalt zu verstehen [5]. Aufgrund verschiedener systemischer Manifestationen wie gastrointestinalen Symptomen werden außerdem begleitende periphere Entstehungsmechanismen vermutet [5].

Die pathophysiologisch zweifelsfrei größte Bedeutung kommt dem Serotoninhaushalt zu, was sich anhand folgender Befunde verdeutlichen lässt:

1. Bei Patientinnen mit PMS wurden, im Vergleich zu gesunden Frauen, geringere Serumserotoninkonzentrationen in der Lutealphase gemessen.
2. Eine diätetische Depletion von Tryptophan, einer Vorläufersubstanz von Serotonin, führt zu einer Exazerbation von PMS-Beschwerden, während die Gabe von Fluoxetin, einem selektiven Serotoninwiederaufnahmehemmer (SSRI), zu einer der effektivsten Therapieoptionen in der Behandlung von PMS und PMDS zählt.
3. Die Applikation von Serotoninantagonisten bei mit Fluoxetin behandelten Patientinnen mit PMS führt zur einem Rezidiv affektiver PMS-Symptome [18–21].

Da durch das Ausschalten der Hypothalamus-Hypophysen-Gonaden-Achse durch die Applikation von GnRH(Gonadotropin-Releasing-Hormon)-Analoga dramatische Verbesserungen von PMS-assoziierten Symptomen erreicht werden können, wird den ovariellen Steroiden eine zentrale Rolle in der Ätiopathogenese von PMS und PMDS zugeschrieben [22, 23]. Aufgrund der Symptommanifestation in der Lutealphase nach Ovulation erscheint es naheliegend, dass veränderte Progesteronspiegel oder ein Ungleichgewicht zwischen der Wirkung von Progesteron und Östrogen an der Entstehung der Symptome beteiligt ist. Allerdings unterscheiden sich die Serumkonzentrationen von Progesteron und anxiolytisch wirkenden Progesteronmetaboliten bei PMS-Patientinnen nicht signifikant von denen nichtbetroffener Kontrollprobandinnen. In vorliegenden Studien führen eine Blockade der Progesteronrezeptoren, z. B. mit dem Progesteronrezeptorblocker Mifepriston, oder die Gabe mikronisierten Progesterons zum Ausgleich des Ungleichgewichts zwischen Östradiol und Progesteron außerdem nicht zu einer signifikanten Besserung der PMS-Symptomatik [5, 24, 25].

Ovarielle Steroide beeinflussen zweifelsfrei, jedoch nicht alleinig, die zyklusabhängige Symptomatik bei PMS und PMDS [5]. Aufgrund der pathologischen Reaktion auf zyklische Hormonveränderungen, die beide Syndrome gemeinsam charakterisiert, werden genetische Polymorphismen spezifischer Hormonrezeptoren als supplementäre, genetische Ursache vermutet [5]. Tierexperimentelle Daten suggerieren, dass zyklische Schwankungen von Östrogen und Progesteron Veränderungen der zentralen β‑Endorphin‑, GABA- und Serotoninkonzentrationen bedingen [5]. Die Bedeutung dieser Befunde für die PMS-Entstehung ist zum aktuellen Zeitpunkt noch weitgehend unklar. Inwieweit beispielsweise der modulierende Effekt von Alprazolam auf PMS-assoziierte Beschwerden tatsächlich auf eine pathophysiologische Bedeutung einer aberranten GABA-Konzentration im ZNS hinweist, verbleibt zum aktuellen Zeitpunkt unklar [26]. Das Auftreten des PMS/PMDS wird zusammenfassend multifaktoriell beeinflusst.

## Differenzialdiagnosen

Die Diagnose und Therapie eines PMS und insbesondere der PMDS ist multidisziplinär. Gynäkologen, Psychiater und ggf. Endokrinologen sollten bei Frauen mit schweren Symptomen involviert werden. Aufgrund der großen Überschneidungen zwischen PMS und affektiven Erkrankungen ist die Differenzierung von PMS und Exazerbation zugrunde liegender psychiatrischer Erkrankungen relevant [10]. Wegweisend ist auch hier, dass sich PMS-assoziierte Symptome in der Lutealphase manifestieren, um nachfolgend in der Follikulärphase zu sistieren, während affektive Symptome sowohl in der Lutealphase als auch in der Follikulärphase bestehen [10]. Eine späte Erstmanifestation PMS-verwandter Symptome in der 5. Lebensdekade sollte eher an eine perimenopausale psychische Alterationen als an eine späte Erstmanifestation eines PMS denken lassen [10]. In der differenzialdiagnostischen Abklärung sollten ferner ovulatorische Störungen mit Lutealinsuffizienz und prämenstruellen Schmierblutungen, persistierende Ovarialzysten und Hyperprolaktinämien berücksichtigt werden. Hormonuntersuchungen der Sexualsteroide, insbesondere bei regelmäßigen Menstruationszyklen und normaler Zykluslänge sind jedoch nicht sinnvoll, da die Symptomatik keine Korrelation mit den Spiegeln der Sexualsteroide, insbesondere nicht mit dem Serumprogesteronspiegel, aufweist. Neben o. g. somatischen Ausschlussdiagnosen stellen affektive Störungen und Angsterkrankungen relevante psychiatrische Differenzialdiagnosen dar [10].

## Evidenz zu Therapie

Während bei milden PMS-Symptomen ohne Funktionsbeeinträchtigung Behandlungsversuche mit Mönchspfeffer, Vitamin B6, Vitamin E, Kalzium, Magnesium, Entspannungsverfahren oder körperlichem Training erwogen werden können, sollte die Behandlung eines diagnosewertigen PMS pharmako- oder psychotherapeutisch erfolgen. Ansätze zur Hormontherapie sind vielfältig und reichen von der zyklischen Gabe mikronisierten Progesterons über die kombinierte orale Kontrazeption, präferenziell im Langzyklus bis zur Zyklussuppression mit GnRH-Agonisten [27]. Die alleinige Gabe von Progesteron ist in Studien nicht ausreichend belegt [27]. Pharmakotherapeutisch sind selektive Serotoninwiederaufnahmehemmer (SSRI) die Behandlungsoption mit den besten Wirksamkeitsbelegen bei schwerem PMS oder PMDS [21, 28]. Alternativ ist der Einsatz eines kombinierten Östrogen-Progesteron-Präparats möglich. Die Behandlung des PMS mit GnRH-Agonisten und Add-back-Therapie ist Fällen mit schwergradig ausgeprägter Symptomatik vorbehalten [28].

Eine Vielzahl von Studien belegt die Wirksamkeit einer SSRI-Behandlung [29–31]. Fluoxetin, Sertralin, Escitalopam und Citalopram sind in dieser Indikation häufig eingesetzte Substanzen [28]. Insbesondere in der Behandlung affektiver PMS-Symptome, aber auch hinsichtlich physischer Symptome und funktioneller Einbußen scheinen SSRI effektiv zu sein [21, 28]. Etwa 60–70 % aller PMS-Patientinnen profitieren von einer SSRI-Behandlung [28]. Bei der SSRI-Therapie besteht die Möglichkeit einer kontinuierlichen Gabe, einer ausschließlichen Lutealphasentherapie und einer rein symptomorientierten Einnahme [21]. Die Lutealphasentherapie wird am 14. Zyklustag begonnen und für gewöhnlich mit Menstruationsbeginn abgesetzt [28]. Sowohl die kontinuierliche als auch die intermittierende Einnahme ist effektiv [21, 32], wobei angemerkt werden muss, dass wenig Vergleichsstudien existieren [21]. Bei schwergradig ausgeprägter Symptomatik scheint die kontinuierliche Gabe wirksamer zu sein [21]. Die SSRI-Dosierungen zur Behandlung des PMS sind vergleichbar mit gängigen Dosierungen in der Behandlung depressiver Störungen [28] und sollten in psychiatrischer Rücksprache festgelegt werden. Die Behandlung erfolgt in den meisten Fällen zunächst für ein Jahr. Nachfolgend wird im Rahmen einer partizipativen Entscheidungsfindung hinsichtlich eines Absetzversuches entschieden [28]. Wird ein kombiniertes Östrogen-Progesteron-Präparat ausgewählt, sollten aufgrund einer der möglichen Verschlechterung affektiver Symptome unter mehrphasigen Präparaten monophasige Darreichungsformen bevorzugt werden [28]. Das antimineralokortikoidwirksame Drospirenon wurde besonders auf die Wirksamkeit bei PMS untersucht. In einer aktuellen RCT zeigte sich die kombinierte Therapie oraler Kontrazeptiva mit Drospirenon und Fluoxetin am effektivsten [33]. Die Evidenz ist insgesamt schmal, metaanalytische Daten zeigen jedoch eine placeboüberlegene Wirksamkeit [34]. Bei schwergradiger Symptomatik, fehlender Wirksamkeit oder Unverträglichkeit von SSRI und Kontraindikationen für östrogenhaltige Kontrazeptiva kann eine Behandlung mit einem GnRH-Agonisten mit Add-back-Therapie erwogen werden [28].

Ferner ist die Evidenz zu psychotherapeutischen Interventionen zum aktuellen Zeitpunkt noch begrenzt. Daten zeigen, dass bspw. eine internetbasierte kognitive Verhaltenstherapie die PMS-assoziierte Symptomlast reduziert [35] und deshalb als therapeutische Option bedacht werden sollte.

## Fazit für die Praxis

- Das PMS und die PMDS sind zyklische Erkrankungsbilder der zweiten Zyklushälfte mit alltagsrelevanter Beeinträchtigung.
- Beide Syndrome können zu einer signifikanten Verschlechterung der Lebensqualität führen.
- Sowohl psychische als auch somatische Symptome prägen das klinische Bild.
- Zu den evidenzbasierten Therapien gehören selektive Serotoninwiederaufnahmehemmer (SSRI), monophasische kombinierte orale Kontrazeptiva und in schweren Fällen GnRH(Gonadotropin-Releasing-Hormon)-Analoga.
